# Methods for genetic manipulation of *Burkholderia gladioli *pathovar *cocovenenans*

**DOI:** 10.1186/1756-0500-3-308

**Published:** 2010-11-16

**Authors:** Nawarat Somprasong, Ian McMillan, RoxAnn R Karkhoff-Schweizer, Skorn Mongkolsuk, Herbert P Schweizer

**Affiliations:** 1 Department of Biotechnology, Faculty of Science, Mahidol University, Ratchathewi, Bangkok, Thailand, 10400; 2 Department of Microbiology, Immunology and Pathology, Colorado State University, IDRC at Foothills Campus, 0922 Campus Delivery, Fort Collins, CO 80523, USA

## Abstract

**Background:**

*Burkholderia gladioli *pathovar *cocovenenans *(BGC) is responsible for sporadic food-poisoning outbreaks with high morbidity and mortality in Asian countries. Little is known about the regulation of virulence factor and toxin production in BGC, and studies in this bacterium have been hampered by lack of genetic tools.

**Findings:**

Establishment of a comprehensive antibiotic susceptibility profile showed that BGC strain ATCC33664 is susceptible to a number of antibiotics including aminoglycosides, carbapenems, fluoroquinolones, tetracyclines and trimethoprim. In this study, we established that gentamicin, kanamycin and trimethoprim are good selection markers for use in BGC. Using a 10 min method for preparation of electrocompetent cells, the bacterium could be transformed by electroporation at high frequencies with replicative plasmids containing the pRO1600-derived origin of replication. These plasmids exhibited a copy number of > 100 in BGC. When co-conjugated with a transposase expressing helper plasmid, mini-Tn*7 *vectors inserted site- and orientation-specifically at a single *glmS*-associated insertion site in the BGC genome. Lastly, a *Himar1 *transposon was used for random transposon mutagenesis of BGC.

**Conclusions:**

A series of genetic tools previously developed for other Gram-negative bacteria was adapted for use in BGC. These tools now facilitate genetic studies of this pathogen and allow establishment of toxin biosynthetic pathways and their genetic regulation.

## Background

*Burkholderia gladioli *pathovar *cocovenenans *was first described in the 1960 s as a bacterium that caused severe cases of food-poisoning and was then named *Pseudomonas cocovenenans *[1]. It has been re-named several times since. In 1995 the bacterium was named *B. cocovenenans *[2,3] and in 1999 it was combined with *B. gladioli*, a phytopathogenic bacterium [4]. Though later analyses confirmed that *B. cocovenenans *and *B. gladioli *should be categorized as a single species, it was deemed necessary to distinguish those strains that are phytogenic from those that also produce toxins lethal to mammals. This resulted in designation of a new pathovar, *B. gladioli *pathovar *cocovenenans *(BGC) in 2003 [5].

Outbreaks of severe BGC-caused food-poisoning are sporadic and seem to be confined to Asia. In endemic regions, fermented coconut can be contaminated with BGC, hence the name "cocovenenans". In particular, bacterial derived toxins have been implicated in deaths resulting from eating the soybean and coconut-based product known as tempe bongkrek. Unlike food-poisoning caused by other bacteria, e.g. *E. coli *O157:H7, mortality rates are significant, reaching > 40% during an outbreak in China in the 1970's [6,7]. Though temporarily called *Flavobacterium farinofermentans *[8], it was later shown that this bacterium was identical to *P. cocovenenans *[9]. Initial symptoms of BGC-caused food-poisoning are evident after a 4-6 hour incubation period and typically include abdominal pains, general malaise, extensive sweating, tiredness and sleepiness, followed by coma. Death usually occurs within 1 to 24 hours after onset of initial symptoms. Diagnosis of the disease is unreliable. This is perhaps best illustrated by a 2007 episode of food-poisoning reported in Central Java (Indonesia)(ProMed Archive numbers 20070802.2439 and 20070806.2557). While this particular episode was first attributed to BGC, this was later doubted and attributed to *E. coli *although the symptoms were clearly more consistent with BGC than *E. coli*.

BGC produces two potent toxins, yellow toxoflavin and colorless bongkrekig acid (BA; also known as bonkrek acid). Though it is not entirely clear whether both toxins contribute to human disease, the evidence points to BA as the major toxin as other bacteria expressing significant levels of toxoflavin, e.g. the rice pathogen *B. glumae*, do generally not cause human disease [10]. A crude toxin preparation (basically supernatant of plate-grown cells) kills mice within 45 min upon oral administration [5]. Toxoflavin production, its biosynthetic pathways and regulatory mechanisms have been studied at the molecular level in *B. glumae *[10]. In contrast, very little is known about toxoflavin and BA production in BGC. A single study showed that BA production is stimulated when BGC is grown in the presence of certain unsaturated fatty acids [11]. Molecular studies in BGC are hindered by lack of genetic tools and in this study we extended the arsenal of tools previously developed for *P. aeruginosa*, *B. pseudomallei *and other Gram-negative bacteria to BGC.

## Results and Discussion

### Determination of antibiotic susceptibility profiles

As most genetic manipulation methods rely on antibiotic selection, it was paramount to determine the antibiotic susceptibility profile of BGC strain ATCC33664 (Table 1). The findings show that this BGC strain is susceptible to aminoglycosides (e.g. gentamicin, kanamycin and streptomycin), carbapenems (e.g. imipenem and meropenem), fluoroquinolones (e.g. ciprofloxacin and norfloxacin), tetracyclines (e.g. doxycycline and tetracycline) and trimethoprim, but quite resistant to many other antibiotics including many β-lactams (e.g. ampicillin, amoxicillin, carbenicillin, and ceftazidime), macrolides (e.g. clarithromycin, clindamycin and erythromycin), chloramphenicol, polymyxin B and zeocin. Many cloning and transposon vectors contain aminoglycoside, tetracycline or trimethoprim resistance selection markers and, because of BCG's susceptibility to the respective antibiotics, should thus be applicable in this bacterium.

**Table 1:** Antibiotic susceptibilities of BGC strain ATCC33664

Antibiotic	MIC (μg/ml)
Ampicillin	64

Amoxicillin	256

Carbenicillin	64

Ceftazidime	32

Imipenem	4

Meropenem	0.75

Ciprofloxacin	0.5

Norfloxacin	8

Clindamycin	> 1024

Clarithromycin	128

Erythromycin	128

Gentamicin	0.125

Kanamycin	0.25

Streptomycin	8

Doxycycline	4

Tetracycline	8

Trimethoprim	0.125

Polymyxin B	> 1024

Zeocin	256

Antibiotic susceptibilities were either determined using E-test (imipenem, meropenem and trimethoprim) or the two-fold serial microdilution method (all others).

### Transformation by electroporation

A prerequisite for any genetic manipulation is the ability to deliver exogenous DNA, e.g. plasmids, into the targeted host strain. The two most commonly used methods are electroporation or conjugation. We previously developed a rapid method for preparation of electrocompetent *P. aeruginosa *cells [12] which was subsequently adapted for use in *B. pseudomallei *[13]. We therefore tested the method in BGC strain ATCC33664. Using the standard method detailed in the Methods section, this strain was subjected to electrotransformation with pUCP28T or pUCP30T. These and other plasmids based on the pRO1600 replicon were previously shown to replicate in other *Burkholderia *species [13,14]. Selection on LB plates containing 100 μg/ml trimethoprim (pUCP28T) or 30 μg/ml gentamicin (pUCP30T) by incubation for two days at 30°C resulted in a transformation efficiency of 10^6 ^and 10^7 ^colony forming units/μg of input DNA which is comparable to efficiencies seen with *P. aeruginosa *[12]. The copy number of pUCP30T in log phase BGC strain ATCC33664 cells growing in LB medium was > 100 as determined by using a previously described quantitative real-time PCR method [13,15]. This is in contrast to *B. pseudomallei *where the copy number of pRO1600 replicon-containing plasmids was determined to be 3-4 [13]. The results indicated that pUCP28T and pUCP30T can efficiently be transformed into BGC, replicate in this bacterium at a fairly high copy number and can thus be used to over-express genes cloned into these plasmid vectors.

### Application of the mini-Tn7 system in BGC

Transposon Tn*7 *based mini-Tn*7 *vectors exhibit several features distinct from plasmid vectors: 1) they integrate into the chromosome site- and orientation-specifically and are thus maintained in single-copy; 2) because they replicate along with the chromosome they are stable and only require antibiotic selection for initial selection of the transposition event; 3) they are broad-host-range and their use is only limited by availability of delivery vehicle transfer method (electroporation or conjugation) and selection marker; and 4) detailed methods for transposon delivery and integration site mapping have been described [16-19].

**Figure 1:**
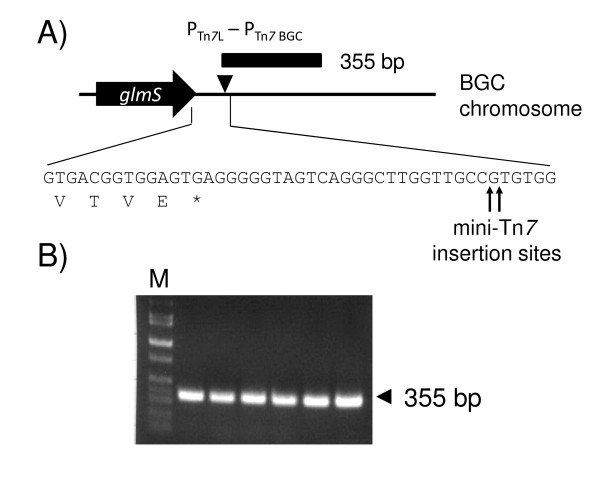
**mini-Tn*7 *insertion site in BGC**. **A) **The mini-Tn*7 *insertion site in BGC strain ATCC33664 determined from rescued plasmids. The sequence shown encompasses the last five codons of a putative *glmS *gene and its immediate downstream sequence, including the mini-Tn*7 *insertion sites 24 and 25 nucleotides downstream of the *glmS *stop codon. **B) **Presence of a single insertion site in BGC. Boiled cell lysates were prepared from six gentamicin resistant transformants obtained after transposition of mini-Tn*7*T-Gm-REP into BGC. Chromosomal DNA contained in boiling preparation lysates was used as a template in PCR reactions containing P_Tn*7*L _and P_Tn*7 *BGC_. The lane labeled M contained Hi-Lo molecular weight markers from Minnesota Molecular (Minneapolis, MN). All other lanes contain PCR products from six gentamicin resistant transformants.

To assess the utility of mini-Tn*7 *based vectors in BGC, the helper plasmid pTNS3 [13] and the mini-Tn*7 *delivery vector pUC18T-mini-Tn*7*T-Gm-REP [18] were transformed into *E. coli *mobilizer strain RHO3 and conjugations with BGC performed as previously described [20]. Co-conjugation of both helper and delivery plasmids into BGC strain ATCC33664 followed by selection on LB agar plates containing 30 μg/ml gentamicin resulted in numerous gentamicin resistant transformants after incubation at 30°C for two days. Because BGC's genome sequence is unknown, we used a previously described method for mapping the Tn*7 *insertion site(s) [17] as detailed in the methods section. Sequencing of DNA flanking the transposon left and rights ends revealed that the insertions occurred either between nucleotides 24 and 25 or 25 and 26 downstream of a putative *glmS *gene (Figure 1). This insertion site and distance from *glmS *is consistent with what has been observed in other bacteria as mini-Tn*7 *elements almost always insert downstream of *glmS*, although rare exceptions do exist [19]. This insertion site was verified in six distinct gentamicin resistant transformants using the BGC-specific primer (P_Tn*7*-BGC_) and a primer annealing to the transposon left end (P_Tn*7*L_)(Figure 1). The expected 355 bp PCR fragment was observed in all six transformants and sequencing confirmed the expected Tn*7 *insertion sites either after nucleotide 24 or 25 downstream of the *glmS *stop codon. Since all *Burkholderia *species analyzed to date contained multiple *glmS *genes and thus multiple mini-Tn*7 *insertion sites [13,16,17], boiling lysates were prepared from 90 gentamicin resistant transformants and used as templates in PCR reactions with P_Tn*7*-BGC _and P_Tn7L_. Observation of the same PCR fragment in all tested transformants indicated a single mini-Tn*7 *insertion site in BGC (data not shown). We were unable to obtain gentamicin resistant transformants in a second transposition event using a strain with an existing insertion at the *glmS*-associated site but having the gentamicin resistance marker deleted by site-specific recombination using Flp recombinase. The overall experimental evidence corroborates the notion of BGC having only one *glmS*-associated mini-Tn*7 *insertion site which can be used for efficient, site-specific insertion of mini-Tn*7 *elements.

### Flp recombinase-mediated marker excision from chromosomally-inserted DNA

Site-specific recombinases, usually yeast Flp recombinase or bacteriophage P1 Cre recombinase, can be used for site-specific excision (deletion) of DNA fragments flanked by the cognate recombination sites, i.e. Flp recombinase target (*FRT*) sites for Flp or *loxP *sites for Cre [21]. We previously demonstrated use of Flp and Cre recombinases in various bacteria, including *B. pseudomallei*, for derivation of markerless mutants [13,22]. Excision of antibiotic selection markers allows recycling of the same resistance determinants in subsequent genetic manipulation procedures. To assess efficiency of Flp recombinase mediated marker excision, we transformed the Flp expression plasmid pFLPe4 into a gentamicin resistant BGC strain containing a chromosomally integrated mini-Tn*7*T-Gm-REP element. On this plasmid, expression of the enhanced Flp structural gene is under control of a rhamnose-regulated promoter [13]. When transformants were plated on LB plates containing 35 μg/ml kanamycin and 0.2% rhamnose, the gentamicin marker was efficiently excised (all kanamycin resistant transformants were gentamicin susceptible). Furthermore, pFLPe4 was rapidly cured from cells grown at 37°C indicating the temperature-sensitive origin of replication contained on this plasmid is functioning at permissive temperature (30°C) but not the non-permissive (37°C) temperature. We conclude that site-specific recombinases can be used in BGC for efficient excision of antibiotic resistance markers from chromosomally integrated DNA elements. Although we have not yet tried Cre recombinase it can be assumed that the Cre/*loxP *system will function in BGC as well.

### Himar1 random mutagenesis

**Figure 2:**
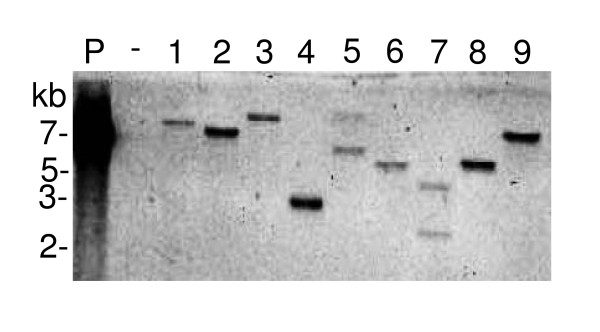
**Random transposition of *Himar1 *in BGC**. Genomic DNA was prepared from nine randomly selected kanamycin resistant transformants, digested for 3 h with *Not*I, and transferred to a nylon membrane. The membrane was hybridized with a probe that detected *ori*_R6K _on the transposon. DNA from BGC strain ATCC33664 was included as a negative control (lane -). The positive control (lane P) was pHBurk3. The 7-, 5-, 3- and 2-kb fragments contained in the biotinylated 2-log DNA ladder (New England Bio-Labs) are indicated on the left. The two hybridizing fragments observed in lanes 5 and 7 are indicative of double *Himar1 *insertions in the respective mutants.

Random transposon mutagenesis is a powerful tool for generation of mutants useful for pathogenesis and other studies. We previously described an efficient *in vivo Himar1 *transposon mutagenesis system for *B. pseudomallei *and demonstrated its use for the isolation of auxotrophic and other mutants [23]. *Mariner*-based transposons function in many bacteria since they do not require host-specific factors and, other than preference for a TA dinucleotide target, do not display target site specificity. To assess the utility of *Himar1 *transposition system in BGC, we transformed pHBurk3 [23] into strain ATCC33664 by electroporation and selected kanamycin resistant transformants at 37°C. At this temperature, pHBurk3 does not replicate and kanamycin resistant colonies should only arise by transposition of the *Himar1 *transposon into chromosomal DNA. Electroporation of 300 ng of pHBurk3 into BGC ATCC33664 yielded 1.6 × 10^4 ^kanamycin resistant colonies at the non-permissive temperature (37°C) and 3.6 × 10^4 ^kanamycin resistant colonies at the permissive temperature (30°C). The calculated rate of transposition is therefore 44%, similar to what has been observed with *B. pseudomallei *[23]. Twenty kanamycin resistant colonies were picked, chromosomal DNA isolated and subjected to genomic Southern analysis using a transposon-specific probe. An analysis of chromosomal DNA from nine colonies is shown in Figure 2. It is evident that the *Himar1 *transposon inserts randomly in chromosomal DNA and there is a low propensity for double insertions. Of a total of 20 colonies analyzed, only three had double insertions (two of them were included in Figure 2, lanes 5 and 7). A preliminary analysis of ~1,700 mutants by replica plating on M9 glucose minimal medium showed a surprisingly low (0.12%) auxotrophy rate. This compares to a rate of 0.72% observed for *B. pseudomallei *[23]. Because the *Himar1 *transposon located on pHBurk3 contains an R6K origin of replication and a kanamycin resistance selection marker, self-ligation of chromosomal DNA fragments obtained by digestion with a restriction enzyme that does not cleave transposon DNA allows rescue of replicating plasmids after transformation of an *E. coli *strain expressing the π protein. Plasmid rescue was performed from one of the auxotrophic mutants and DNA sequencing revealed that the insertion occurred within the amidophosphoribosyltransferase structural gene *purF*. Nutritional supplementation experiments with adenine restored growth of the mutant on M9 glucose minimal medium and supported the functional assignment of *purF *within the *de novo *purine biosynthetic pathway. The experimental evidence shows that the *Himar1 *transposon can be used in BGC for generation of random chromosomal mutations.

## Conclusions

We have shown that many of the tools previously developed for other *Burkholderia *species function efficiently in BGC and can thus be used for genetic manipulation of this bacterium, and probably the closely related phytopathogenic *B. gladioli*. Determination of the antibiotic susceptibility profiles indicated that aminoglycosides (gentamicin and kanamycin) as well as trimethoprim resistance markers provide a clean selection in BGC using relatively low antibiotic levels. This was initially demonstrated by successful electroporation of two replicative plasmids, pUCP28T and pUCP30T, using a rapid, microcentrifuge-based procedure for preparation of electrocompetent cells. Co-conjugation of a helper and delivery plasmid from *E. coli *to BGC showed that mini-Tn*7 *elements can be readily transposed into the BGC genome. Unlike other *Burkholderia *species, BGC seems to contain only one *glm*S-linked insertion site. Transposition events can readily be monitored by developments of a BGC-specific primer and defined PCR conditions. Site-specific excision of the gentamicin resistance marker from the chromosomally-integrated mini-Tn*7 *element using Flp recombinase proved the functionality of the Flp/*FRT *system for marker excision in BGC and rapid curing of the Flp expression plasmid at non-permissive temperature. Lastly, we established that the *mariner *based *Himar1 *transposon can be used for rapid, random mutagenesis of the BGC genome. The tools and methods described here provide the basis for genetic manipulation and modification of BGC, something that was to date unachievable. The authors realize that the conclusions relating to the applicability of the methods to the species are based on a single isolate. However, a limited number of BGC strains are available and it is likely that most studies will be performed using the type strain, ATCC33664, used in this study.

## Methods

### Bacterial strains, media and growth conditions

*B. gladioli *pathovar *cocovenenans *(BGC) ATCC33664 was obtained from the American Type Culture Collection, Manassas, VA. *E. coli *strains used were DH5α(λ*pir*)(laboratory collection) and RHO3 [20]. RHO3 is a Δ*asd *mutant and requires 400 μg/ml diaminopimelic acid (DAP; LL-, DD-, and meso-isomers; Sigma, St. Louis, MO) for growth on rich media. Bacteria were routinely grown at 37°C (*E. coli*) or 30°C (BGC) in Luria broth Lennox (LB) [24] or on LB agar purchased from MO BIO Laboratories, Carlsbad, CA. Strains containing temperature-sensitive (TS) plasmid derivatives or TS alleles were grown at 30°C (permissive temperature) or 37°C (non-permissive temperature). M9 medium [25] with 10 mM glucose was used as the minimal medium and supplemented with 0.6 mM adenine to support growth of *purF *mutants. Antibiotics were added at the following concentrations: 100 μg/ml ampicillin, 15 μg/ml gentamicin and 35 μg/ml kanamycin for *E. coli*; 15-30 μg/ml gentamicin and 35 μg/ml kanamycin for BGC. Minimal inhibitory concentrations (MICs) were determined in Mueller-Hinton broth (Becton Dickinson, Franklin Lakes, NJ) by the 2-fold broth microdilution technique following Clinical and Laboratory Standards Institute guidelines [26]. The MICs were recorded after incubation at 30°C for 24 h. Antibiotics were purchased from the following manufacturers: carbenicillin was from Duchefa Biochemie via Gold Biotechnology, St. Louis, MO; ciprofloxacin was from LKT Laboratories, St. Paul, MN; gentamicin was from EMD Biosciences, San Diego; zeocin was from Invitrogen, Carlsbad, CA; and all others were from Sigma, St. Louis, MO.

### DNA and genetic methods

Details of most of the methods described herein have previously been described and adapted for use in BGC with minor modifications.

### Plasmids

Plasmid DNAs were isolated from *E. coli *and *Burkholderia *spp. by using a Fermentas GeneJET Plasmid MiniPrep Kit (Fermentas, Glen Burnie, MD). Plasmids used in this study included the replicative pUCP28T [14], pUCP30T [14] and pFLPe4 [13]. The non-replicative plasmids pTNS3 [13], pUC18T-mini-Tn*7*T-Gm-REP [18] and pHBurk3 [23] were used as sources for the Tn*7 *site-specific transposition pathway, mini-Tn*7*T-Gm-REP, and *Himar1 *transposon, respectively. The GenBank accession numbers for plasmids are U33751 for pUCP28T; U33752 for pUCP30T; EU215438 for pFLPe4; EU215432 for pTNS3; AY712952 for pUC18T-mini-Tn*7*T-Gm-REP and EU919403 for pHBurk3.

### Electrocompetent cells, transformation and plasmid copy number

Bacterial cells were made competent by a rapid method [12]. Briefly, cells were grown overnight at 30°C and 1 ml of this culture was harvested by centrifugation in a microfuge at 8,000 rpm for 2 min. The cells were washed twice with 1 ml of sterile 300 mM sucrose by resuspension with a pipet tip and centrifugation at 8,000 rpm for 2 min. Cells were finally resuspended in 100-200 μl of 300 mM sucrose. All solutions and manipulation steps were at room temperature. One hundred microliter aliquots of electrocompetent cells were then used in electroporation experiments using previously described conditions, except that after electroporation cells were incubated at 30°C for 1 h prior to plating on selective media. BGC cells were typically transformed with 100 to 200 ng of plasmid DNA. A quantitative real-time PCR method was used for determination of plasmid copy number in BGC [13,15]. With this method, the separate detections of a plasmid and host chromosomal DNA were achieved using two separate primer sets, specific for the plasmid aminoglycoside resistance gene (*aacCI*) and for the chromosomal, single-copy aspartate-β-semialdehyde dehydrogenase gene (*asd*). Primers were designed using Primer 3 software and were as follows: for *aacC1*, aacC1-Q-F (5'-CTGATGTTGGGAGTAGGTG) and aacC1-Q-R (5'-GTTAGGTGGCTCAAGTATGG), yielding an 130-bp product; and for *asd*, asd-RT-UP (5-ACACGTCGTTCGTGTAGTCG) and asd-RT-DN (5 AAAACGAGACCACGCTCAAG), yielding a 99-bp product. Real-time quantitative PCR amplifications of the total DNA samples from two separate BCG ATCC33664 cultures harboring pUCP30T were performed. The absolute copy numbers of *aacC1 *and *asd *genes in the BGC total DNA samples were determined from the respective standard curves, using the threshold cycle values. The plasmid copy numbers of pUCP30T was calculated by dividing the absolute copy number of *aacC1 *by the copy number of *asd*. Experiments were repeated three times for calculation of copy number.

### Transposition of mini-Tn7 and determination of insertion site

The helper plasmid pTNS3 [13] and the mini-Tn*7 *delivery vector pUC18T-mini-Tn*7*T-Gm-REP [18] were transformed into *E. coli *mobilizer strain RHO3 [20]. Co-conjugation of both helper and delivery plasmids into BCG strain ATCC33664 was achieved by triparental mating. *E. coli *and BGC cultures were grown overnight at 37°C or 30°C, respectively. 200 μl of each culture was transferred to the same microcentrifuge tube and cells harvested by centrifugation at 8,000 rpm at room temperature. Cells were washed twice with LB medium and then resuspended in 30 μl of LB. The cell suspension was transferred to a filter sitting on an LB agar plate containing 400 μg/ml DAP. After overnight incubation at 30°C, the filter was transferred to a microcentrifuge tube containing 1 ml of LB and cells dislodged by vortexing for 60 s. Aliquots (50 and 100 μl) were spread on LB-agar plates with 30 μg/ml gentamicin and plates incubated at 30°C for two days. For insertion site mapping, we used a previously described method [17]. Briefly, six gentamicin resistant transformants were grown overnight in LB medium with 30 μg/ml gentamicin and genomic DNA was isolated using a PUREGENE^® ^genomic DNA purification kit (Gentra Systems, Minneapolis, MN). 1 μg of this DNA was then digested with *Not*I for 2 h and the resulting DNA fragments were self-ligated overnight at 14°C. The ligation mixtures were transformed into DH5α(λ*pir*) and gentamicin resistant transformants selected by plating on LB medium with 15 μg/ml gentamicin. Plasmid DNA was then isolated from transformants grown in LB medium with 15 μg/ml gentamicin and sequenced using either P_Tn*7*L _or P_Tn*7*R _as a primer. After insertion site mapping, a BGC-specific primer (P_Tn*7*-BGC_; 5'-GGAACAGGCTGAAACGAGAG) was used in conjunction with P_Tn*7*L _(5'-ATTAGCTTACGACGCTACACCC;[18]) to rapidly identify transformants with mini-Tn*7 *insertions at this *glmS*-associated site by colony PCR using DNA in boiling preparation lysates as templates. These lysates were obtained by transferring separate colonies to individual sterile microcentrifuge tubes containing 30 μl of sterile H_2_O and boiling for 5 min. Insertions at this site are characterized by amplification of a 355 bp DNA fragment (Figure 1B).

### Flp mediated excision of antibiotic selection markers and curing of expression plasmid

Flp-mediated excision of chromosomally inserted gentamicin resistance markers was achieved using previously described procedures and pFLPe4 [13]. Briefly, cells were transformed with 150 ng pFLPe4 DNA and kanamycin resistant cells selected at 30°C on LB plates with 35 μg/ml kanamycin and 0.2% rhamnose to promote FLP gene expression. Kanamycin resistant transformants were struck for single colonies onto the same selective plates and colonies were then patched on LB and LB + 30 μg/ml gentamicin plates, followed by incubation at 30°C. Gentamicin susceptible colonies indicate successful excision of the gentamicin resistance marker. For curing of pFLPe4, a gentamicin susceptible colony was inoculated into LB medium and grown overnight at 30°C. Cells from this culture were then struck onto LB agar plates for single colonies and the plates incubated at 37°C. Single colonies growing on these plates were then patched onto LB, LB + 30 μg/ml gentamicin and LB + 35 μg/ml kanamycin plates. Cells growing only on the LB plates were saved.

### Himar1 transposition into BCG and insertion site mapping

pHBurk3 is a *Himar1 d*elivery plasmid with a temperature sensitive origin of replication, a *Himar1 t*ransposon containing a kanamycin resistance selection marker and an R6K-derived origin of replication [23]. For transposition into BGC, electrocompetent cells of strain ATCC33664 were transformed with 300 ng of pHBurk3 DNA and kanamycin resistant colonies selected at 37°C on LB plates containing 35 μg/ml kanamycin. Kanamycin resistant colonies growing at this temperature represent cells containing chromosomally inserted *Himar1 *transposons. For genomic Southern analysis, 20 kanamycin resistant colonies were picked, grown overnight in LB medium with 35 μg/ml kanamycin and genomic DNA isolated with the Gentra PUREGENE^® ^DNA purification kit. DNA (2 μg) was digested with *Not*I for 3 h, electrophoresed on a 1% agarose gel, and transferred to positively charged nylon membranes (Roche Diagnostics Corp., Indianapolis, IN) by passive transfer as previously described (23). Following transfer and UV fixation, blots were probed with a PCR fragment biotinylated by random hexamer priming following the NEBlot Phototype labeling and detection kit protocols (New England BioLabs, Beverly, MA). The probe detected the *HimarBP *transposon with a 376-bp fragment recognizing the *ori*_R6K _region.

## Competing interests

The authors declare that they have no competing interests.

## Authors' contributions

NS performed most of the experiments, IM determined plasmid copy numbers, RKS designed and supervised experiments, SM participated in student supervision, and HPS designed and supervised experiments and drafted the final manuscript. All authors read and approved the final manuscript.
